# TT2020 meeting report on the 16th Transgenic Technology Meeting

**DOI:** 10.1007/s11248-020-00231-w

**Published:** 2021-01-21

**Authors:** Reetta Hinttala, Satu Kuure

**Affiliations:** 1 Medical Research Center Oulu and PEDEGO Research Unit, University of Oulu and Oulu University Hospital, Oulu, Finland; 2 Biocenter Oulu, University of Oulu, Oulu, Finland; 3 GM-Unit, Laboratory Animal Center, Helsinki Institute of Life Science, University of Helsinki, Helsinki, Finland; 4 Stem Cells and Metabolism Research Program, Faculty of Medicine, University of Helsinki, Helsinki, Finland

**Keywords:** ISTT, Transgene, Genome editing, International conference, Israel, Virtual meeting

## Abstract

The 16th transgenic technology (TT) meeting of the International Society of Transgenic technology (ISTT) took place on October 26–29th 2020 and was quite unique as it was the first-ever virtual meeting in the history of ISTT events. Dr. Rebecca Haffner-Krausz at Weizmann Institute of Science, Israel, was the local organizer of the meeting, which attracted 756 registered participants from 32 different countries.

## Introduction

**Fig. 1:**
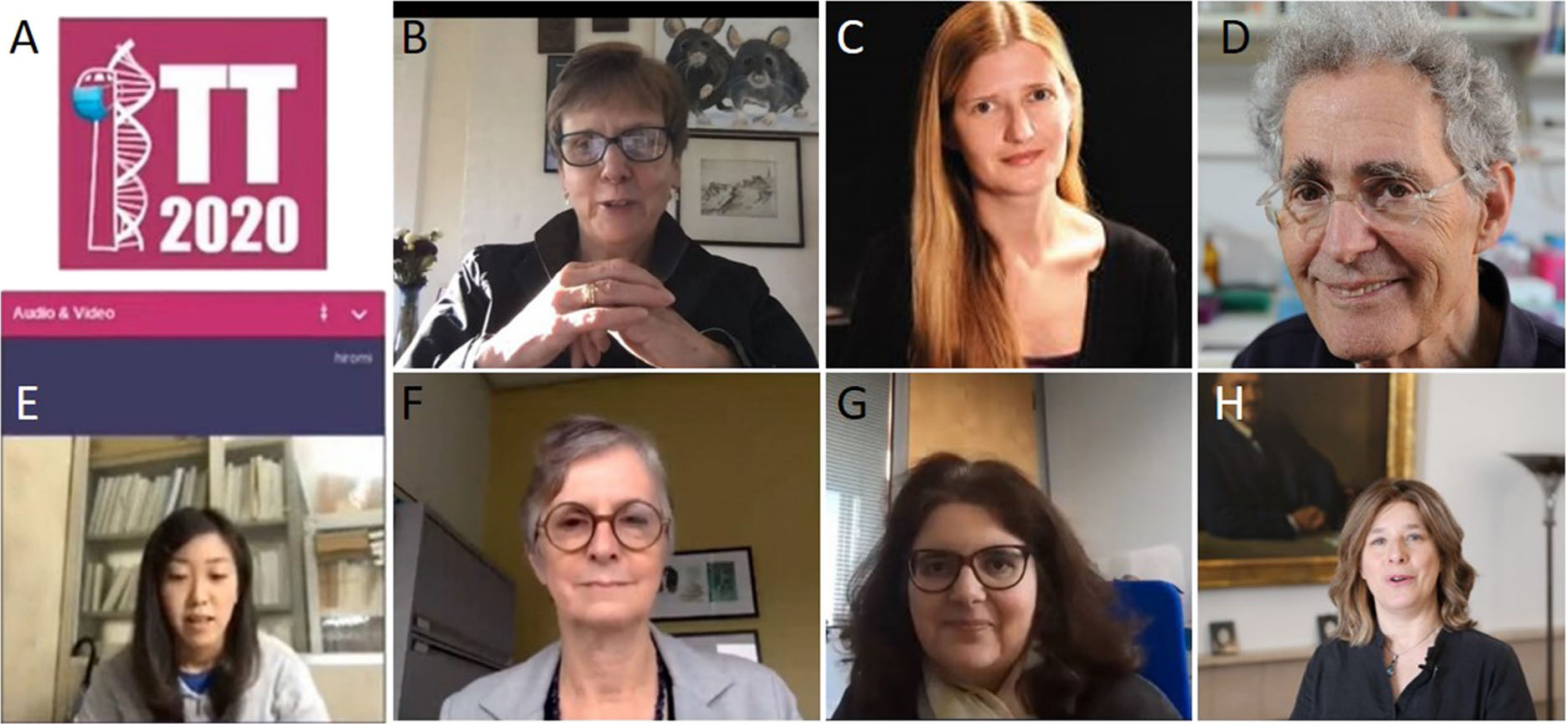
The different prize winners and main organizer of TT2020. **a** An elegant logo of TT2020, which was originally planned to take place in Israel, but eventually turned into virtual meeting, was modified with the mask to capture the existing pandemic. **b** The ISTT Prize 2020 was awarded to Dr. *Alex Joyner* from Sloan Kettering, New York, USA. **c** The Orbis Pictus Awardee 2020 is Dr. *Elizabeth Hillman* from Columbia University, New York, USA. **d** Dr. *Howard Cedar* (The Hebrew University of Jerusalem, Israel) gave a keynote lecture on remote control of gene expression by DNA methylation in the Epigenetics session. **e** The Young Investigator Award 2020 was presented to Dr. *Hiromi Miura* from Tokai University School of Medicine, Japan. **f** Dr. *Janet Rossant* (SickKids, Toronto, Canada) gave the EMBO keynote lecture on dynamics of early development. **g** The 3R prize, was awarded to Dr. *Lydia Teboul* from MRC Harwell, UK. **h** The main organizer of TT2020, Dr. *Rebecca Haffner*-*Krausz* (Weizmann Institute of Science, Israel)

The TT2020 was originally planned as a regular on-site international meeting, where the society members and those who are interested in animal genetic engineering gather together to share their latest technological and scientific highlights. The global pandemic of Covid-19 changed the plans for TT2020 similarly as for many other events, and it was timely turned into virtual meeting. This quite doubled the workload for the main organizer Dr. Rebecca Haffner-Krausz (Fig. 1) and her team (Table 1) who succeeded with the help of the board of directors to put together very well-functioning and amazingly interactive TT2020 under such a challenging situation.

**Table 1:** The organizing team of TT2020

Name	Organization	Role
Rebecca Haffner-Krausz	Dept Vet Resources, Weizmann Institute of Science, Israel	Chairperson, Conference Organizer
Daniela Levine	Dept Vet Resources, Weizmann Institute of Science, Israel	Secretary
Inbal Azoulay	Conference Centre, Weizmann Institute of Science, Israel	Administration
Ella Melamed	Kenes, Israel	Virtual Platform Coordinator
Simoul Interactive	Israel	Virtual Meeting Platform Development

### Day 1

#### Session 1

Panel Discussion—Animal Models: From Bench to Bedside.

Was chaired by Karen Avraham (University of Tel Aviv, Israel) and Jan Parker-Thornburg (MD Anderson Cancer Center, USA). In this session, sponsored by Genetic Engineered Mouse Models (GEMM), National Israel Facility, experts in the field of gene-editing and disease modeling presented their latest advancements in developing treatment options for human diseases.

The first speaker of the session was *Paula Rio* from CIEMAT (Research Centre for Energy, Environment and Technology, Madrid, Spain) who is interested in developing Gene Therapy for Fanconi anemia and was talking about how to transfer research from the lab to the clinic.

*Eithan Galun* (The Hebrew University of Jerusalem, Israel) talked about disease mechanisms leading to therapeutic approaches of combined hepatocellular carcinoma—cholangiocarcinoma (cHCC-CCA) using the MDR2 KOFoxl1CRE; RosaYFP mice as a model organism.

*Aris Economides* (Regeneron, USA) introduced his studies aiming at development of antibody-based therapy for an ultra-rare skeletal disorder called fibrodysplasia ossification progressive, which is caused by mutations in activin A receptor, type 1 gene. His presentation underlined the importance of an accurate animal disease model for testing the anti-activin A blocking antibody effects prior the option to use any new therapeutic intervention in patients will be realistic.

*Tom Wishart* (Roslin Institute, UK) discussed encouraging examples of utilizing livestock model systems in translating novel therapeutic approaches for neurological conditions to successful clinical applications. In his talk, he brought up an example of the first CRISPR generated ovine model of CLN1 form of Batten disease in order to improve the understanding of the human condition and to provide a model for assessing therapeutic efficacy.

#### Session 2

Epigenetics.

The second session chaired by Janet Rossant from The Hospital for Sick Children (Toronto, Canada) was dedicated for the recent discoveries of epigenetics aided by CRISPR/Cas9 genome editing. The session represented well the excellence in Israeli researchers as four out of the total five speakers in this session were local and grantees of many prestige awards.

The session was opened by *Howard Cedar* (The Hebrew University of Jerusalem, Israel, Fig. 1) one of the world leading scientists in the area of DNA methylation and epigenetic control. He gave a keynote lecture on remote control of gene expression by DNA methylation. In his talk, Dr. Cedar focused on epigenetic signals during development providing an annotation system that operates on a network of genome-wide activator and repressor elements that control how and when genes are read in the organism both during normal development and as an adaptive response to environment and behavior.

The second speaker of the session was *Wei Li* (Chinese Academy of Sciences, China), who is interested in differences between the parental genomes and utilized haploid embryonic stem (ES) cells in his studies of genomic imprinting. His research indicates that genetic modification of multiple gene simultaneously is easier in haploid than normal ES cell lines.

Next, *Eran Meshorer* (The Hebrew University of Jerusalem, Israel) demonstrated how comparative epigenetics can be used as a powerful tool in revealing the genetic basis of ancient humans, present-day humans, and chimps based on differentially methylated genomic regions. Especially genes such as NFIX, associated with face and vocal tract anatomy exhibit extensive methylation changes through evolution. In his talk, Dr. Meshorer demonstrated the generation and analysis of NFIX Tg mice showing significant changes in vocalizations.

*Yonatan Stelzer* (Weizmann Institute of Science, Israel) highlighted the local research activities by his presentation that focused on computational modeling mouse of gastrulation at single embryo and single-cell resolution. They have developed an algorithm to annotate kinetics and developmental trajectories in gastrulating embryos and validated it with the use knockout ES cells and single embryo chimera.

The final speaker of the session *Nissim Benvenisty* (The Hebrew University of Jerusalem, Israel) talked about generation of haploid human embryonic stem cells from unfertilized human oocytes. He demonstrated the power of haploid human ES cells in genetic screening by generating a genome-wide loss-of-function library utilizing the CRISPR/Cas9 technology. Using this library, they characterized essential genes of each cellular compartment in promoting or restricting cell growth and categorized human genetic disorders according to their role in early embryogenesis.

The first day ended with the presentation by the *ISTT Prize winner* (Fig. 1), who was introduced by the ISTT president Wojtek Auerbach. The 2020 Prize, kindly and generously sponsored by Genoway, was awarded to Dr. *Alex (Alexandra) Joyner* (Sloan Kettering, New York, USA) who received not only outpouring of virtual congratulations but also the prize itself, the silver mouse blastocyst figure, in a virtual form during the broadcasting. In her award lecture entitled “Genetic exploration of neural stem cell self-renewal and plasticity” Dr. Joyner first gave glimpses of her career that have sculptured her as a scientist, showed very concrete examples of the early experiments and their documentation, as well as homemade equipment like PCR and electroporation machines. She acknowledged specifically Dr. Gail Martin and Janet Rossant, both of them past ISTT Prize winners, as key persons in her career and Wojtek’s role as a key person who established the required gene targeting techniques at New York University. In the scientific part of the talk Joyner focused on the contributions her lab has made by genetic labeling experiments to advance the understanding of cell fates and temporal gene functions and requirements in neurogenesis.

### Day 2

The second day started out with concurrent sessions on large animal and non-mammalian transgenesis and continued with ever-emerging important ethics presentations and discussions.

#### Sessions 3a

Large animal transgenesis.

Chaired by Bruce Whitelaw (University of Edinburgh, UK) began with studies focusing on early development and interspecies organogenesis and then shifted towards utilization of transgenic animals in food and therapeutic applications.

*Jun Wu* (UT Southwestern Medical Center, USA) talked about derivation of intermediate pluripotent stem cells (PSCs) amenable to primordial germ cell (PSC) specification. Dr. Wu presented a novel method for derivation of PSCs from mouse and horse blastocysts and from transgene-free induced pluripotent stem cells from horse and human fibroblasts that are valuable for studying mammalian pluripotency and early PGC development.

*Hui Yang* (Chinese Academy of Sciences, China) presented striking experimental data on generation of functional rat cell-reconstituted forebrain in mice using an improved blastocyst complementation method. He showed how their approach in injection of rat embryonic stem cells into the mouse blastocyst in which organogenesis-relevant gene was deleted by CRISPR editing enabled rapid screening of organogenesis-relevant genes and allowed efficient interspecies organogenesis.

*Alison Van Eenennaam* (UC Davis, USA) talked about agricultural animal transgenesis for food applications. In particular, she addressed the challenges in commercialization of genetically engineered (GE) food animals. As an example, only a single GE food animal, the fast-growing AquAdvantage salmon, has been commercialized to date even though the first GE livestock were reported already 35 years ago. Dr. Van Eenennaam suggested that a cost:benefit analysis should be incorporated into regulatory decisions about transgenic and gene edited livestock to make evident the opportunity costs of precluding the adoption of safe breeding innovations.

At the end of the session, *Nathan Buzzell* (LFB USA, inc.) introduced transgenic goats that express high levels of recombinant Trastuzumab, which is a recombinant monoclonal antibody therapeutic approved for treating both breast and stomach cancer. In summary, Dr. Buzzell demonstrated that their technology platform is a robust and cost effective system for production of Trastuzumab over multiple lactations and generations of the transgenic goat line.

#### Session 3B

Non-mammalian transgenesis.

The ever-expanding non-mammalian model organisms session was chaired by Richard Behringer (University of Texas M.D. Anderson Cancer Center, Houston, USA) and included presentations about ants, worms, cuttlefish and snails. The session had good gender balance among the speakers and great representation from the USA. The discussion related to questions and answers was right after the talks, which worked nicely and promoted spontaneous and lively interactions.

*Claude Desplan* (New York University, New York, USA) started the session by presenting his work with insect visual system and entitled “Manipulating aging”. In his presentation, Dr. Desplan used CRISP to produce ants that cannot smell pheromones and showed experiments that suggest that insulin through its activities in ovarian growth regulation controls lifespan in ants.

*Swathi Arur* (MD Anderson Cancer Center, Houston, USA) addressed a fundamental question of how maternal nutrition supports fetal development with transgenic and CRISPR/Cas9 analysis in *C. elegans*. Interestingly, CRISPRing in *C. elegans* requires injections, as it cannot just be bombarded with reagents and for each gene editing, approximately 20 worms are injected followed by four-to-five founders are used for breeding and phenotype analysis in the next generations.

*Tessa Montague* (Columbia University, New York, USA) is a postdoc from Richard Axel’s lab who reminded the audience about the expansion of model organisms and showed nice BBC video of how cuttlefish change their color according to the environmental changes. Such accommodation to habitation is dependent on brain function and one of her aims is to utilize transgenic cuttlefish expressing GCaMP in neurons to enable simultaneous visualization of skin color change and neuronal activation to understand how brain functions deconstruct the visual context to skin color adaptation.

*Tim Corbin* (Stowers Institute, Kansas City, USA) described what does it take to make transgenic snails. Early snail embryo is very difficult to manipulate as it is very sticky—a property, which has to be removed before its genetic manipulation is possible. He also needed to find ways to increase rigidity of an early snail embryo and visualize reagent introduction in its very dark cytoplasm before successful genome editing with good efficiency was possible in snail.

#### Session 4

Ethics discussion by panelists—Ethics of Gene Editing in Animals.

The traditional ethics session chaired by Lluis Montoliu completed the second day of meeting program. The three presentations in this session were given by excellent scientists, who have vast experience of ethical framework in animal experiments and genome editing.

*Lluis Montoliu* (National Centre of Biotechnology, Madrid, Spain), focused on ethical considerations of rapidly developing genome editing techniques. While modern editing tools are constantly improved to be more efficient in generating mutant mice compared to the standard approaches, the number of animals used remains the same. However, the possibility to introduce mutations efficiently in very complex genetic backgrounds, carrying already numerous other mutant alleles, can save many animals from traditional breeding schemes. Importantly, Dr. Montoliu emphasized the importance of adequate on-target validation rather than simply focusing on off-targets.

*Kirk Leech* from The European Animal Research Association (EARA) was the second speaker of the session and started out by explaining what EARA is and what are its strategic aims. The talk was entitled “Time to talk” and he then accordingly focused on how to improve awareness, openness and transparency of animal research. A simple example of openness is to provide animal research statement in the institution’s webpage to inform the public that animal research is carried out within this institution. There have been small improvements in openness during the previous years, but that they have been rather modest and the vast majority of institutes do not clearly acknowledge animal research at their institutes.

*Andy Greenfield* (MGU, Harwell, Oxford, UK) discussed the recent progress of a working group of the UK Nuffield Council on Bioethics in examining the ethics of genome editing in farmed animals. Potential applications in farmed animals promise improved food security and higher standards of animal welfare. However, these proposed interventions in farming practices exist in a social, ethical and political space that is highly contested and require open discussion.

### Day 3

#### Session 5

Germ Cells, IPS and Trophoblast Cells.

Chaired by Yonatan Stelzer (Weizmann Institute of Science, Israel) began by studies focusing to understand the detailed mechanisms behind reprogramming and cell fate.

*Nicolas Rivron* (Austrian Academy of Science, Vienna) showed how trophoblast and embryonic stem cells self-organize in vitro to form structures called blastoids that morphologically and transcriptionally resemble blastocysts. Specifically, Dr. Rivron demonstrated how embryonic cells maintain trophoblast proliferation and self-renewal, while fine-tuning trophoblast epithelial morphogenesis. He concluded that blastoids are a powerful tool that can be generated in large numbers, finely tuned, contain all the cell types to form the conceptus, and implant in utero.

*Yossi Buganim* (Hebrew University-Hadassah Medical School, Israel) talked about comparative parallel multi-omics analysis of cell undergoing reprogramming. Their analysis revealed that cells undergoing reprogramming to induced pluripotent stem cells and induced trophoblast stem cells exhibit specific trajectories from the onset of the process, suggesting ‘V’-shaped model. As a conclusion, they identified novel stage-specific reprogramming markers and markers for faithful reprogramming and reprogramming blockers.

*Nitzan Gonen* (Bar-Ilan University, Israel) presented her research on sex development/determination and its disorders. Dr. Gonen is specifically interested in modeling human XX genotype associated with WT1 mutations in mouse. Patients with these mutations show testicular tissue formation and some are phenotypically assigned as males and some females. Mouse models had to be characterized as embryos because of the lethality at late gestation, but they recapitulate some of the patient phenotype well as they show masculinization of the fetal gonads.

The session continued by the *ORBIS PICTUS Lecture*, which is sponsored by the Czech Centre for Phenogenomics. The deputy director of the Phenogenomics, Jan Rozman introduced the awardee of 2020, who was *Elizabeth Hillman* (Fig. 1.) from Columbia University (NY, USA). Dr. Hillman gave an inspirational, pre-recorded talk on “Visualizing in vivo systems at the speed of life” and was available online afterwards to participate in lively discussion her presentation evoked. She walked the audience through the many different imaging systems she as a physicist has developed and applied on live imaging of extremely diverse biological phenomena. The talk finished by thanking the transgenic community for generation of fluorescent models that enable live imaging.

#### Session 6

Roundtable—Running a Transgenic Unit.

The session chaired by Peter Hohenstein (Leiden University Medical Center, The Netherlands) was running very smoothly, despite of the missing camera/video connection in the beginning. The roundtable discussion had representatives from academic core facility leaders (Peter, Karen Brennan, Australian National University, Australia and Ben Davis, Oxford University, UK), technical staff (Lisbeth Ahm Hansen, Aarhus University, Denmark) and commercial service provider (Philip Damiani, Charles River, US), and turned out interesting and informative. The discussion was inspired by four core facility specific poll questions in the beginning and which were then used as starting points for further argumentation. The whole session went very well in a controlled manner and dealt with issues such as electroporation vs microinjection of CRISPR-reagents, impact of Covid-19 pandemic on core facility services, and possibility to implement new technologies in different core facilities. As a final, thoughtful detail; the roundtable topic discussions started in the session were continued in the following session’s social hour tables.

### Day 4

#### Session 7

3Rs.

Was chaired by Branko Zevnik (University of Cologne, Germany). The program of the session covered different aspects of 3Rs by introducing technical approaches to reduce and refine the number of animals used in research and to enhance the reproducibility of biomedical studies.

*Ivo Huijbers* (Netherlands Cancer Institute, The Netherlands) presented strategies for fast and flexible mouse model generation with GEMM-ESC strategy that utilizes ES cells from the previously available mouse models. His examples were from studies related to small cell lung cancer and expanded to non-germline strategy for modeling breast cancer with BRCA1-mutations in mouse.

The second speaker of the session was *Sara Wells* (MRC Harwell, UK). She turned our attention to latest developments in phenotyping refined and sophisticated new mouse strains. In her presentation she showed how continuous automated home cage assessment is particularly relevant for investigating novel models and progressive conditions where the phenotypes are unpredictable, subtle or their onset is hard to reliably observe through traditional out of cage phenotyping methods.

Traditional *3R prize*, 3rd in row and generously sponsored by Janvier Labs, was awarded to Dr. *Lydia Teboul* (MRC Harwell, UK) for her efforts in development of 3R principles in animal research (Fig. 1). She expressed her gratitude for this prize as they have worked hard to develop methods to identify correct allele editing in complex allelic background, which allows the best possible founder selection for breeding and thus has practical impact on the number of animals used.

At the end of the session, *Young Investigator Award* was presented to *Hiromi Miura* (Fig. 1) by Benoit Kanzler (Max Planck Institute of Immunobiology and Epigenetics, Germany). The prize was established to identify and recognize young researchers who keep the field vital and attractive. This year the prize was supported by an independent medical education grant from Regeneron Pharmaceuticals, Inc.

*Hiromi Miura* (Tokai University School of Medicine, Japan), the Young Investigator 2020 Awardee, presented her impressive research history related to the development of several breakthrough transgenic technologies utilized by researchers worldwide including Pronuclear Injection-based Targeted Transgenesis (PITT), Efficient additions with ssDNA inserts-CRISPR (Easi-CRISPR), and improved-Genome-editing via Oviductal Nucleic Acids Delivery (i-GONAD).

#### Session 8

Emerging Technologies.

The session on emerging technologies chaired by Soren Warming (Genentech, San Francisco, USA) showcased an impressive repertoire of new applications for CRIPRS/Cas9 and touched upon an important phenome of microbiota’s effect on translatability of animal research.

*Kimberly Cooper* (University of California, San Diego, USA) addressed the differences in female and male germline double strand break mechanisms by copycat transgenesis in combination with Cas9 expression in Spo11 locus. Her results suggest that the improved rate of super-Mendelian inheritance requires more robust and earlier Cas9 expression than what Spo11 locus provides.

*Stephan Rosshart* (University Medical Center Freiburg, Germany) gave an interesting talk about limitations of laboratory mice with divergent microbiota to predict the complex immune responses of humans. To address the challenge, they transferred C57BL/6 embryos into wild mice, creating “wildlings.” These mice combine the natural microbiota of wild mice with the tractable genetics of lab mice. Importantly, wildlings, but not conventional laboratory mice, phenocopied human immune responses in two preclinical studies.

*Daniel Schramek* (Mount Sinai Hospital, Toronto, Canada) challenged the traditional way of genomics and proteomics research in cell lines and takes them in his own research to in vivo mouse models to better address association vs causality of human mutations. This utilizes CRISPR/Cas9-based method to generate thousands of knockout libraries in specific organs (e.g. skin, mammary gland, brain, lung or pancreas) to identify tumor suppressor genes in given organ.

*Jessica Woodley* (IDT, Integrated DNA Technologies, USA) gave IDT-sponsored talk where she presented their work demonstrating that improved efficiency in CRISPR-based homology-directed repair (HDR) rates for large insertions is obtained when dsDNA donor templates include novel end-modifications. These modifications improved the frequency of HDR and reduced homology-independent insertion events.

#### Session 9

Animal models for COVID-19 Research.

Was chaired by Lauryl Nutter (The Centre for Phenogenomics, Canada) and focused on the predominant topic of the current era. After two presentations describing ferrets and hamsters as model organisms to understand the mechanisms behind and to treat SARS-CoV-2, the session ended with poster awards sponsored by Charles River.

*Sander Herfst* (Erasmus University Medical Center, Rotterdam, The Netherlands) is interested in how much of the SARS-CoV-2 virus transmissions occurs via air and presented his results in ferrets. Dr. Herfst’s data showed that three out of four ferrets were infected without direct contact with virus when the distance was 1 m and two out of four when the distance is longer. In the end of his talk, Dr. Herfst specifically underlined that these results have no direct extrapolation to humans as ferrets and mustelids are very susceptible for SARS-CoV-2 infection.

*Benjamin tenOever* (Icahn School of Medicine at Mount Sinai, USA) discussed the systemic inflammation response to SARS-CoV-2 both in humans and in hamster model and a distinct transcriptional footprint comprising of high chemokines and low interferons (IFN) signature caused by the viral infection. Altogether, the talk provided a molecular basis for the symptoms encompassing infection and support the use of intranasal IFN-I as an effective prophylactic against SARS-CoV-2.

### EMBO keynote lecture

Karen Avraham (University of Tel Aviv, Israel) had the honor to introduce the keynote lecturer and the last speaker of the TT2020 conference *Dr. Janet Rossant* (The Hospital for Sick Children,Toronto, Canada) (Fig. 1), a true trailblazer in the field of developmental biology and a winner of numerous prestigious prizes including the 10^th^ ISTT prize.

*Janet Rossant* gave a thorough overview on dynamics of early development. By studying the mouse blastocyst and its derived stem cells, they have identified distinct signaling pathways and transcription factors that specify pluripotent versus extraembryonic cell fate. Even though tools of single cell genomics and live imaging provide new insights, there is still a lot to learn about stem cell states and lineage relationships across species.

### E-posters and poster awards

There were two E-poster sessions on day 2 and day 3. Over 50 E-posters were presented, and they covered the animal models, 3Rs, new developed techniques, and many general issues. The *ISTT best poster* committee chaired by Martina Crispo and assisted by Aimee Stablewski, Benoit Kanzler, Geraldine Schlapp, Jorge Pórfido and Yael Goldfarb chose the three Charles River-sponsored poster prize awardees, who were introduced by Christopher Dowdy (Charles River, TX, USA). The prize for the new technologies was given to Steven Bischoff (NovoHelix, FL, USA), for the animal models to Irena Jenickova (Czech Center of Phenomics, Czech Republic) and for the optimization to Andrew Syvyk (Blinn College, TX, USA).

## Conclusions

It was hard to predict in advance, what the outcome of the first-ever virtual TT2020 meeting would be. Impressive number of participants from all over the world were registered to the meeting (Table 2). Almost half of the participants were not ISTT members (353) highlighting clearly the influential power and research quality of transgenic technologies also in scientific communities outside of the society. The daily average of participants logging into the sessions was around 550 reflecting the nature of virtual meetings, which allows smooth selections of presentations one wants to follow.

**Table 2:** TT2020 demographics

Total # registered participants	756
ISTT member participants	403
Countries represented	32
Sponsors	31
Exhibition booths	16
Abstract submissions	58
Poster presentations	56

We were amazed about the efforts the organizers had made in boosting interaction among the speakers, board members, society, sponsors and participants. The virtual look of the meeting place itself was truly stunning with the possibilities to “walk in” to different lecture halls, meet the sponsors at their booths and “sit down” to different tables during the social hour sessions. The vast majority of the sessions with presentations were running extremely well and after some technical and practical issues during the first social and poster sessions also the interactive parts of the meeting started to run smoothly.

We were very honored to reveal the next TT meeting place in the end of the last meeting day. TT2022 will be organized as a hybrid on-site/virtual meeting in an exotic Levi ski resort in Norther Finland. Many vital lessons were learnt from TT2020 that will help organizing the hybrid meeting together with the commercial *In Conferences* being onboard for the first time. We are very much looking forward to the challenge of TT2022 organization and welcoming you all to participate—either via high-quality virtual connection or preferably in person in Levi, Finland!

